# Increased tauopathy drives microglia-mediated clearance of beta-amyloid

**DOI:** 10.1186/s40478-016-0336-1

**Published:** 2016-06-23

**Authors:** Wesley Chen, Edsel A. Abud, Stephen T. Yeung, Anita Lakatos, Trevor Nassi, Jane Wang, David Blum, Luc Buée, Wayne W. Poon, Mathew Blurton-Jones

**Affiliations:** Department of Neurobiology & Behavior, University of California Irvine, Irvine, CA 92697 USA; Sue and Bill Gross Stem Cell Research Center, University of California Irvine, Irvine, CA 92697 USA; Institute for Memory Impairments and Neurological Disorders, University of California Irvine, 845 Health Science Rd, 3200 Gross Hall, Irvine, CA 92697 USA; Present Address: Department of Immunology, University of Connecticut School of Medicine, Farmington, CT 06030 USA; University Lille, Inserm, CHU-Lille, UMR-S 1172, Alzheimer & Tauopathies, Lille, France

**Keywords:** Amyloid, Aβ, Tau, Mice, Phagocytosis, Cytokines, Microglia, Pathology

## Abstract

Alzheimer disease is characterized by the accumulation of β-amyloid (Aβ) plaques and tau-laden neurofibrillary tangles. Emerging studies suggest that in neurodegenerative diseases, aggregation of one protein species can promote other proteinopathies and that inflammation plays an important role in this process. To study the interplay between Aβ deposition, tau pathology, and microgliosis, we established a new AD transgenic mouse model by crossing 5xfAD mice with Thy-Tau22 transgenic mice. The resulting ‘T5x’ mice exhibit a greater than three-fold increase in misfolded and hyperphosphorylated tau and further substantiates the hypothesis that Aβ accelerates tau pathology. Surprisingly, T5x mice exhibit a 40-50 % reduction in Aβ plaque load and insoluble Aβ species when compared with aged-matched 5xfAD littermates. T5x mice exhibit significant changes in cytokine production, an almost doubling of microglial number, and a dramatic shift in microglia activation state. Furthermore, T5x microglia exhibit increased phagocytic capacity that enhances the clearance of insoluble Aβ and decreasing plaque load. Therefore, our results suggest that strategies to increase the phagocytic ability of microglia can be employed to reduce Aβ and that tau-induced changes in microglial activation state can promote the clearance of Aβ.

**Electronic supplementary material:**

The online version of this article (doi:10.1186/s40478-016-0336-1) contains supplementary material, which is available to authorized users.

## Introduction

Alzheimer’s Disease (AD) is the leading cause of age-related dementia, affecting over 5 million people in the United States alone [5]. AD pathology is characterized by two primary lesions: extracellular amyloid plaques and intraneuronal tau-laden neurofibrillary tangles. The mechanisms that drive AD remain unclear, but the ‘amyloid cascade hypothesis’ first proposed by Hardy and Higgins posits that beta-amyloid (Aβ) accumulation is the initiating factor in AD pathogenesis [8, 31, 33]. Increased deposition of Aβ, in turn, is thought to promote the hyperphosphorylation of tau leading to neurofibrillary tangle (NFT) formation [2]. Together, Aβ and tau pathologies disrupt critical biological functions such as axonal transport and synaptic function and promote neuroinflammation, ultimately leading to widespread synaptic and neuronal loss [65, 83].

The role of neuroinflammation in the development and progression of AD has been studied for several decades [26, 27, 34]. However, the recent identification of AD risk polymorphisms in several microglial-enriched genes such as TREM2, MS4A, and CD33, has intensified this area of research [9, 15, 25, 28, 32, 42, 51, 86]. Microglia serve as one of the brain’s primary mechanisms of Aβ clearance, but also play critical roles in neuronal homeostasis and synaptic plasticity [63, 69, 76]. In response to Aβ, microglia increase their expression of pro-inflammatory cytokines, which has been shown to promote tau hyperphosphorylation and NFT pathology [10, 49] and contribute to synaptic and neuronal dysfunction [3, 30, 69].

To better understand the role of microglia in the interactions between Aβ and tau pathologies we crossed two transgenic AD models, 5xfAD and Thy-Tau22 mice, to create a novel bigenic line termed ‘T5x’ mice. 5xfAD mice express three APP mutations (Swedish, Florida, London) and two PS1 mutations (M146L, L286V) that are co-inherited and driven under control of the neuronal-specific Thy1.2 promoter [61]. 5xfAD exhibit intraneuronal Aβ accumulation beginning at 1.5 months, amyloid plaque deposition and gliosis starting at 2 months, synaptic loss by 6 months, and neuronal death beginning at 9 months of age [22]. Amyloid deposition in these mice is most prominent in the hippocampus, subiculum, deep cortical layers, and the basal lateral amygdala. Increased neuroinflammation and microglial activation have also been shown to play an essential role in mediating disease progression in this model [43]. Thus, 5xfAD mice provide an aggressive amyloidogenic model that exhibit robust AD-associated plaque pathology and microgliosis. In comparison, Thy-Tau22 mice express human four repeat tau with two mutations (G272V, P301S) driven under the Thy1.2 promoter and progressively develop hippocampal hyperphosphorylated tau, neurofibrillary tangles, and gliosis [72]. Studies examining the effects of exercise, caffeine and A_2A_ receptor modulation in Thy-Tau22 mice suggest important roles for microglia and neuroinflammatory responses in the accumulation of tau pathology [7, 44].

Interactions between Aβ and tau in mouse models were first reported in two seminal studies in 2001 that clearly demonstrated that Aβ accumulation could accelerate the development of tau pathology [24, 47]. Subsequently, the 3xTg-AD model was generated and extended our understanding of the influence of Aβ on tau and was used to explore the role of microglial inflammation in this process [39, 62]. Since then several other bigenic models have been created and studies have continued to investigate the effects of amyloid on tau and suggested that the amyloid cascade follows a unidirectional pathway [18, 35, 71]. However, many of these models exhibit far less pathology than occurs in human AD cases and thus more complex interactions that might occur over decades in the human brain or at later stages of advanced disease may not be faithfully recapitulated in many of these models [85]. Furthermore, the potential role of inflammation in these interactions between Aβ and tau remains greatly understudied.

In the present study, we combined a model of Aβ accumulation (5xfAD mice) with a progressive model of neurofibrillary tangle pathology (Thy-Tau22 mice). The resulting ‘T5x’ mice were generated to provide insight into the later stages of disease progression akin to that observed in clinically-diagnosed AD patients and the interactions and consequences of advanced amyloid pathology on tau and visa-versa. By comparing T5x bigenic mice to their single transgenic littermates we have uncovered potential new roles for tau in the modulation of Aβ and neuroinflammatory response. Most notably, we identify and examine how amyloid and tau synergize to alter microglial activity and promote Aβ clearance. Thus, these data provide additional insight into the relationship between AD pathology and neuroinflammatory response and suggest that tau can exhibit reciprocal interactions with amyloid.

## Material and Methods

### Generation of T5x mice

Thy-Tau22 mice express human 4 repeat tau with two frontotemporal dementia-associated point mutations (G272V and P301S) under control of the neuronal driven promoter Thy1.2 and are maintained on a C57Bl6/J background [72]. The 5xfAD mice used in this study are also maintained on a congenic C57Bl6/J background and were purchased from the Mutant Mouse Resource and Research Center (MMRRC, stock# 034848-JAX). The 5xfAD model co-expresses human amyloid precursor protein (APP695) carrying the Swedish, Florida, and London mutations and a human presenilin-1 (PS1) transgene carrying the M146L and L286V mutations under the Thy-1 promoter. Both APP and PS1 transgenes are co-integrated and thus co-inherited. Heterozygous Thy-Tau22 and 5xfAD mice were crossed to create Thy-Tau22-5xfAD (T5x) mice, as well as Thy-Tau22, 5xfAD, and WT littermates that were genotyped via PCR amplification of human tau, PS1, and APP transgenes. The number of mice from each sex-balanced genotype was: WT (*n* = 8), 5x (*n* = 11), Tau (*n* = 14), and T5x (*n* = 9). All mice were maintained on a purebred C57Bl/6 background and group housed (2–4 mice/cage) on a 12 h/12 h light/dark cycle with access to food and water ad libitum. All animal procedures were performed in strict accordance to the National Institutes of Health and University of California Institutional Animal Care and Use Committee.

### Tissue preparation and neuropathological analysis

Following behavioral testing, all mice were sedated with euthasol and sacrificed by cardiac perfusion with 0.1 M phosphate buffered saline (PBS). Brains were removed and one hemisphere was snap frozen on dry ice while the other hemisphere was postfixed in 4 % paraformaldehyde for 48 h then stored in PBS + 0.05 % sodium azide. Fixed half-brains were placed in 30 % sucrose for at least 48 h before being cut in the coronal plane (40 μm sections) using a freezing sliding microtome.

### Immunohistological staining

Brain sections were rinsed in PBS three times and blocked in PBS + 0.05 % Triton-X with 5 % donkey or goat serum for one hour. Primary antibodies used included: 6E10 (Covance; 1:500) and 82E1 (ABL America; 1:500) against Aβ, total Tau (human + mouse; Dako; 1:1000), human tau (HT7, ThermoFisher; 1:1000), phosho-tau epitopes AT8 (Pierce; 1:500) and PHF-1, and conformational tau epitope MC-1 (generously provided by Peter Davies; 1:1000). Analysis of gliosis, phagocytosis, and dendritic architecture utilized CD68 (Abcam; 1:200), IBA1 (Wako; 1:1000), GFAP (Abcam; 1:1000), Beta3Tubullin (Covance; 1:1000). Sections were incubated in primary antibodies at 4° Celsius overnight. Sections were then washed three times with PBS and incubated with appropriate Alexa fluor-conjugated secondary antibodies at room temperature for one hour. Sections were then rinsed three additional times, mounted on slides and coverslipped with Fluoromount-G with DAPI.

### Biochemical analysis

Hippocampus and cortex was microdissected from frozen brains and processed to collect both soluble and insoluble extracts. Briefly, microdissected tissue was homogenized in TPER (ThermoFisher) and centrifuged at 12,000 RPM for 15 min. Supernatant was collected as the soluble fraction and the pellet was treated with 70 % formic acid and spun down at 25,000 rpm. The resulting supernatant was collected as the insoluble fraction. Insoluble protein samples were neutralized for Western blotting and further precipitated with trichloroacetic acid (TCA) when probing for insoluble tau. Protein samples were denatured at 95 °C for 15 min before being loaded onto 4-20 % TGX precast polyacrylamide gels (Bio-rad). Antibodies used for western blotting include: HT7 (1:1000), PS199 (Abcam; 1:1000), PS202 (Abcam; 1:1000), AT100 (ThermoFisher; 1:1000), AT270 (ThermoFisher; 1:1000), PHF1 (1:1000), 6E10 (1:1000), GFAP (1:1000). Mesoscale Discovery immunoassay kits (Mesoscale Diagnostics) were used for cytokine (K15048G) and Aβ38, 40, and 42 (K15199E) quantification of cortical samples following the manufacturers protocols. The proinflammatory MSD was able to detect levels of that were within the standard curve, whereas brain levels of IFN-γ, IL-12p70, CXCL1 and IL-4 were below the threshold of detection.

### Confocal microscopy and quantification

Equivalent brain sections were picked and immunofluorescent sections were imaged using Olympus FX1200 confocal microscope. Amyloid plaque burden identified by 82E1 were visualized through a Z-stack image taken through the entire depth of the section at 1 μm intervals. Confocal files were then rendered in 3D and analyzed by a blinded observer using the volume function of IMARIS software (Bitplane). Microglia and astrocyte quantification by confocal microscopy was also analyzed using IMARIS. Microglia number was quantified using IMARIS spot function and process morphology was measured using filament length and branching functions. Astrocyte IMARIS quantification was performed using the volumetric function. Microglia phagocytosis of Aβ was quantified using a combined immunofluorescent staining of IBA1, 82E1, and CD68. High magnification Z-stack images were taken of randomly selected plaques while being blinded to IBA1 microglial staining. Quantification of internalized Aβ was done according to previously described protocols [40, 54]. To account for varying total microglia numbers across images, the internalized Aβ index was normalized to the number of microglia per image. Quantification of neuronal degeneration was performed in equivalent hippocampal brain sections using immunohistochemical labeling of Beta-III Tubulin (B3T). Four randomly selected square sub-areas were selected in pyramidal, radiatum, or molecular layers of hippocampus CA1 and quantified by optical density using ImageJ.

### Nanostring analysis

RNA was isolated from microdissected hippocampi of WT, Thy-Tau22, 5xfAD, and T5x mice using RNeasy Plus Universal Mini Kit (Qiagen). RNA samples were run a custom Nanostring panel (Nanostring Technologies) examining mouse AD-linked genes. To evaluate mouse amyloidogenesis, we compared levels of murine RNA for genes APP, BACE1, BACE2, ADAM10, PSEN1, and PSEN2.

### Statistical analysis

Statistical analysis was performed using StatView software (SAS Institute Inc.). Statistical comparisons between multiple groups were performed using ANOVA followed by Fischer’s PLSD post-hoc tests. Error bars represent the standard error of the mean. Groups were considered statistically significant when *p < 0.05 for both ANOVA and posthoc analysis.

## Results

### Aβ pathology induces robust tau hyperphosphorylation and neurofibrillary tangle formation in T5x mice

**Fig. 1:**
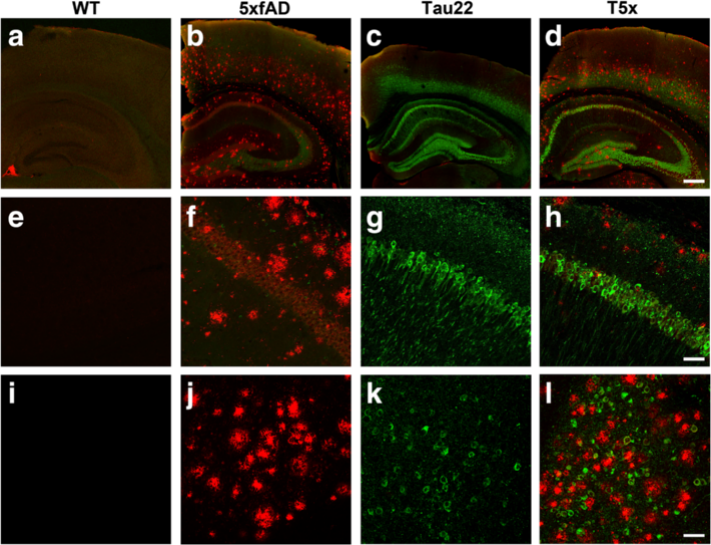
T5x mice exhibit robust accumulation of both beta- amyloid and tau pathologies within the hippocampus, cortex, and amygdala. Tau (Green; Dako total tau) and beta-amyloid (Red; 6E10) were examined by immunofluorescence and confocal microscopy in half brain coronal sections, revealing appropriate labeling of Aβ plaques and/or tau-laden neurofibrillary tangles in the hippocampus and overlying cortex of each genotype; WT (**a**), 5xfAD (**b**), Thy-Tau22 (**c**), T5x (**d**). Confocal settings were first established using T5x mice and then identical settings were used for all genotypes. Higher power images of the CA1 region of the hippocampus and amygdala further demonstrate a lack of either pathology in WT controls (**e**, **i**), Aβ pathology in 5xfAD mice (**f**, **j**), tau pathology in Thy-Tau22 mice (**g**, **k**), a combination of amyloid and tau pathology in T5x (**h**, **l**). Scale Bar = 300 μm in (**a**-**d**), 50 μm in (**e**-**g**), and 100 μm in (**i**-**k**)

**Fig. 2:**
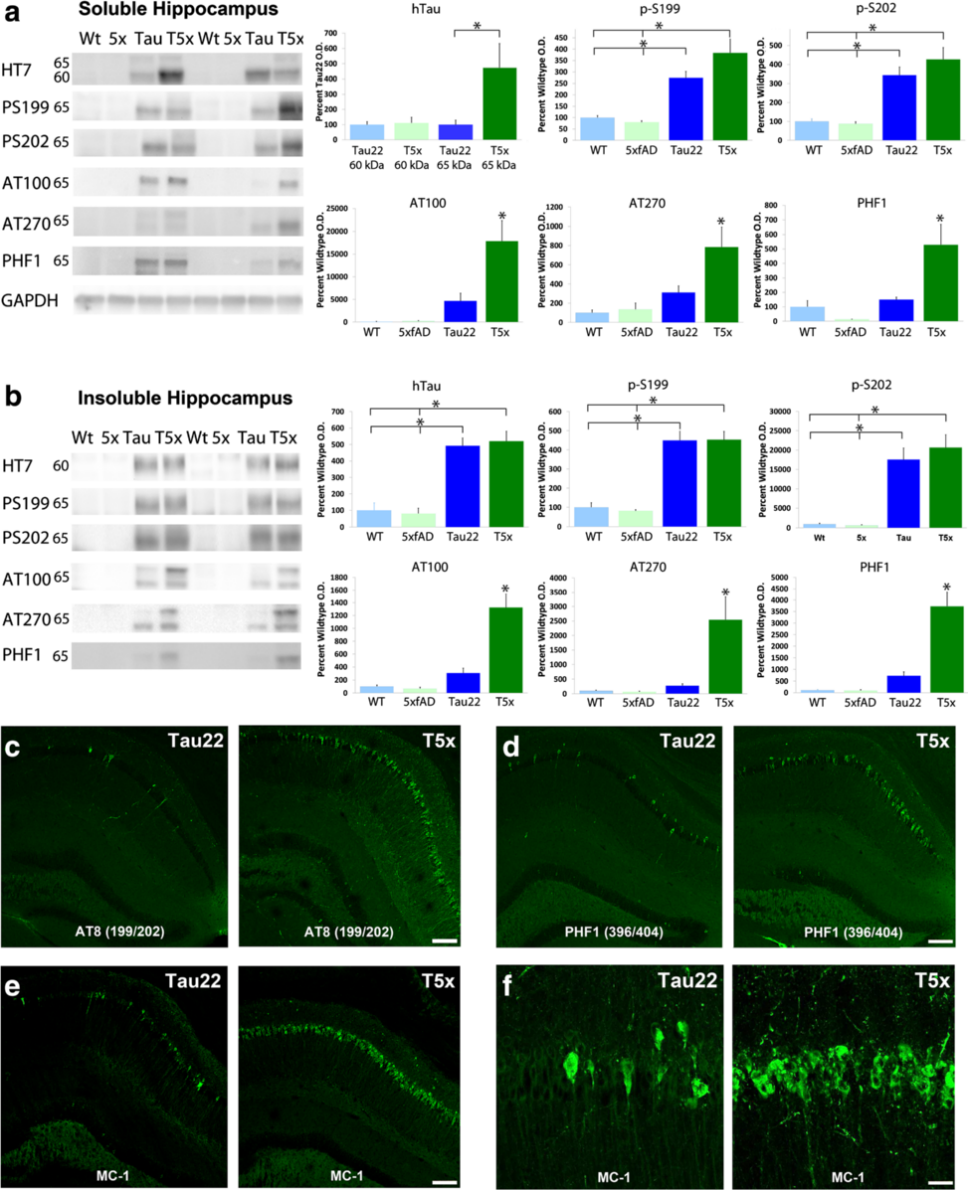
Hippocampal tau hyperphosphorylation is greatly increased by concurrent beta-amyloid pathology. Western blot and immunofluorescent analysis was used to examine the effects of concurrent Aβ and tau pathology on tau accumulation, phosphorylation, and solubility. Examination of the soluble fraction of microdissected hippocampi (**a**), revealed an electrophoretic shift in total human tau (HT7) to produce a second band at 65 kDa, likely representing hyperphosphorylation of tau (*p* = 0.0007), but no significant change in the unphosphorylated band at 60 kDa (*p* = 0.71). Western blotting for several tau phosphoepitopes revealed similar 60 and 60 kDa bands. In this case, as both bands represent phosphoryalation of that epitope, quantification was performed on the combined bands. While phosphorylation at Ser199 and Ser202 was unchanged between Thy-Tau22 and T5x mice, several other pathological phospho-epitopes including AT100 (*p* = 0.0002), AT270 (*p* = 0.0002), and PHF1 (*p* = 0.003) exhibited robust 2–4 fold increases in T5x hippocampal fractions versus Thy-Tau22 lysates (**b**). Whereas total levels of insoluble human tau (HT7) and Ser199 and Ser202 epitopes were unchanged between T5x and Thy-Tau22 mice, T5x mice exhibited dramatic 7–10 fold increases of insoluble AT100, AT270, and PHF1 phosphorylated tau (*p* < 0.0001). Representative immunohistochemical labeling of AT8 (**c**) and PHF1 (**d**) immunoreactive tangles in the hippocampus further illustrate the enhancement of tau hyperphosphorylation induced by Aβ pathology in T5x mice. Labeling for the tau conformational epitope MC1 at both low (**e**) and high (**f**) power magnification likewise reveals a large increase in MC-1 immunoreactive CA1 neurons (quantification shown in Additional file 2: Figure S2). Data are represented as mean ± SEM, normalized to % of WT group, *n* ≥ 8 mice/group. * Indicates *p* < 0.05 for both ANOVA and Fisher’s protected least-significant difference (PLSD) post hoc tests with significance versus all other groups, whereas *over a bar indicates significance between 2 or 3 particular groups. Scale Bar = 100 μm in (**c**-**e**) and 50 μm in (**f**)

Brains of WT, Thy-Tau22, 5xfAD, and T5x mice (7 months) were examined by immunocytochemistry to assess accumulation of tau and beta-amyloid. Robust accumulation of tau (green) and Aβ (red) plaques was detected throughout the brain of T5x mice with extensive extracellular amyloid plaque pathology and intraneuronal tau accumulation observed within the hippocampus, neocortex and amygdala (Fig. 1). Next, biochemical approaches were used to quantify changes in tau pathology induced by Aβ accumulation. Soluble levels of total human tau (HT7 antibody) were quantified in the hippocampus and cortex of 7-month old littermates of each genotype (Fig. 2, Additional file 1: Figure S1). As expected, human tau was detectable only in mice that expressed the human tau transgene; Thy-Tau22 and T5x. Interestingly, levels of the 60 kDa human tau species were not significantly different between T5x and Thy-Tau22 littermates in either the hippocampus (*p* = 0.33) or cortex (*p* = 0.13). In contrast, the HT7 antibody also recognized a higher molecular weight tau species (65 kDa) that exhibited marked increases in T5x versus Thy-Tau22 mice in both the hippocampus (*p* = 0.0007; Fig. 2a) and cortex (*p* = 0.01; Additional file 1: Figure S1A). The ratio of the phosphorylated 65 kDa tau band to the unphosphorylated 60 kDa tau band was also significantly higher in T5x mice relative to Tau22 littermates in hippocampus (*p* = 0.004; Additional file 2: Figure S2A) and cortex (*p* = 0.001; Additional file 2: Figure S2A). Because the hyperphosphorylation of tau results in an electrophoretic shift in migration, these data provide evidence that Aβ accumulation leads to increased soluble hyperphosphorylated tau in T5x mice.

To further characterize how Aβ alters tau hyperphosphorylation, soluble hippocampal and cortical lysates were probed for multiple pathological tau epitopes (i.e. AT100, AT270, PHF-1). For many of the phospho-epitopes, two bands were often detected, likely representing tau species with varying degrees of hyperphosphorylation at multiple sites. Quantification of these phospho-epitopes within both the hippocampus and cortex, demonstrate that many exhibit a 2–3 fold elevation in T5x mice compared to Thy-Tau22 littermates (Fig. 2, Additional file 1: Figure S1, *p* < 0.001). As tau becomes hyperphosphorylated, it aggregates and becomes increasing insoluble. Therefore, we examined whether insoluble tau was also increased within T5x mice. While total levels of insoluble human tau (HT7) were unchanged between T5x and Thy-Tau22 mice, T5x mice exhibited a dramatic increase in several hyperphosphorylated insoluble tau species, as AT100, AT270, and PHF1 levels were elevated 5–8 fold in T5x mice compared to Thy-Tau22 littermates (Fig. 2, Additional file 1: Figure S1, *p* < 0.001).

Increased tau hyperphosphorylation within T5x brain was further confirmed immunohistochemically. The brains of T5x mice exhibited increased numbers and density of AT8, AT100, and PHF1 immunoreactive neurons within both the hippocampus and cortex when compared to Thy-Tau22 (Figs. 2c-d, Additional file 1: Figure S1C-D). In AD, it is hypothesized that tau misfolds prior to hyperphosphorylation and this change can be detected with the conformation-specific antibody MC-1 [38]. Indeed, MC-1 immunoreactivity was more prevalent in the brains of T5x versus Thy-Tau22 mice, exhibiting a more than three-fold increase in the hippocampus and cortex (Figs. 2e-f, Additional file 1: Figure S1E-F and 2B). Thus, consistent with previous reports, it appears that the presence of beta-amyloid pathology accelerates the accumulation, misfolding, and hyperphosphorylation of tau in T5x mice.

### T5x mice exhibit differential levels of cytokines in brain

**Fig. 3:**
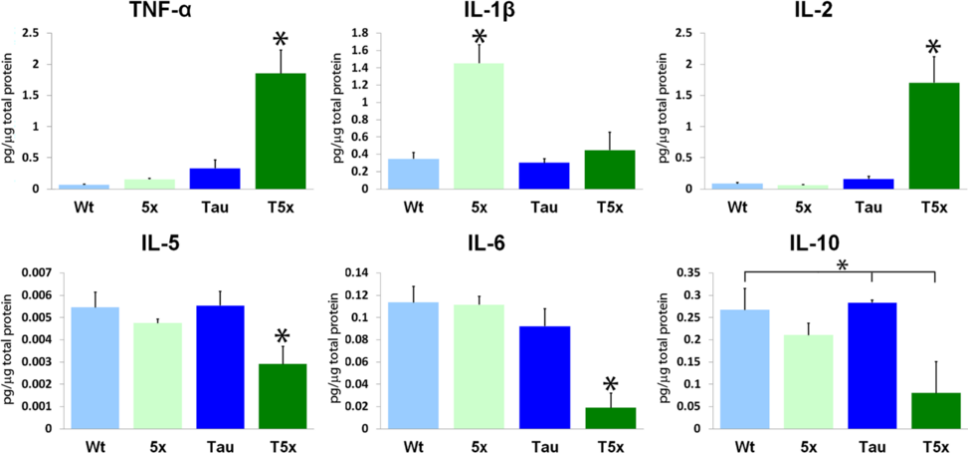
The combination of Aβ and tau pathology alters the production of cytokines within the brain. MSD multiplex ELISA was utilized to examine several key cytokines. Compared to WT, Tau and 5xfAD littermates, T5x mice showed a significant increase in both TNF-α and IL-2 (*p* < 0.0001). In contrast, T5x mice exhibited a significant decrease in IL-1β in comparison to 5xfAD littermates (*p* < 0.0001) as well as decreases in IL-5, IL-6, and IL-10 (*p* < 0.05). Data are represented as mean ± SEM, *n* ≥ 8 mice/group. * Indicates *p* < 0.05 for both ANOVA and Fisher’s protected least-significant difference (PLSD) post hoc tests with significance versus all other groups, whereas *over a bar indicates significance between 2 or 3 particular groups

Cytokines have been implicated in the modulation of microglia number and function in the presence of beta-amyloid and tau pathologies. To determine the combined effects of tau and amyloid pathology on neuroinflammation, we quantified the protein levels of IL-10, IL-1β, IL-2, IL-5, IL-6, and TNF-α within the cortex (Fig. 3). In T5x mice, a surprising three-fold decrease in IL-1β relative to 5xfAD mice was observed (*p* < 0.0001; Fig. 3). Interestingly, IL-5, IL-6, and IL-10 levels were also decreased in T5x mice compared to 5xfAD littermates (*p* < 0.05; Fig. 3). In contrast, T5x mice showed an approximately 7-fold increase in both IL-2 and TNF-α compared to wild-type and transgenic littermates (*p* < 0.0001). Because IL-2 and TNF-α levels highly correlate with PHF-1 (Additional file 3: Figure S3, IL-2: PHF-1 R^2^ = 0.481, TNF-α: PHF-1 R^2^ = 0.557), this suggests that increasing tau pathology likely plays a role in the induction of these cytokines. Whereas IL-2 and TNF-α promotes microglial activation and proliferation, IL-10 has been shown to inhibit microglial Aβ phagocytosis [19, 29, 57, 89]. Taken together, the cytokine profile of T5x mice suggested that microglial function might be differentially affected by varying amounts of tau and amyloid and led us to investigate whether microglia number or activation state were also altered in T5x mice.

### Tau and Aβ synergistically modulate astrocyte and microglia number and morphology

To determine whether neuroinflammatory changes in T5x mice were associated with astrogliosis, astrocyte morphology was examined in all genotypes (Additional file 4: Figure S4A). Astrocyte total volume, as assessed by GFAP immunoreactivity, was increased 2–3 fold in T5x mice compared to 5xfAD and Thy-Tau22 littermates within CA1 and the cortex (Additional file 4: Figure S4B, ANOVA and PLSD *p* < 0.05). Western blot analysis of cortical fractions further corroborated these results revealing an approximately two-fold increase in GFAP protein levels in T5x lysates (Additional file 4: Figure S4C, ANOVA and PLSD *p* < 0.05). This increase in astrogliosis is suggestive of a synergistic immune response to the combination of tau and beta-amyloid pathology in T5x mice.

**Fig. 4:**
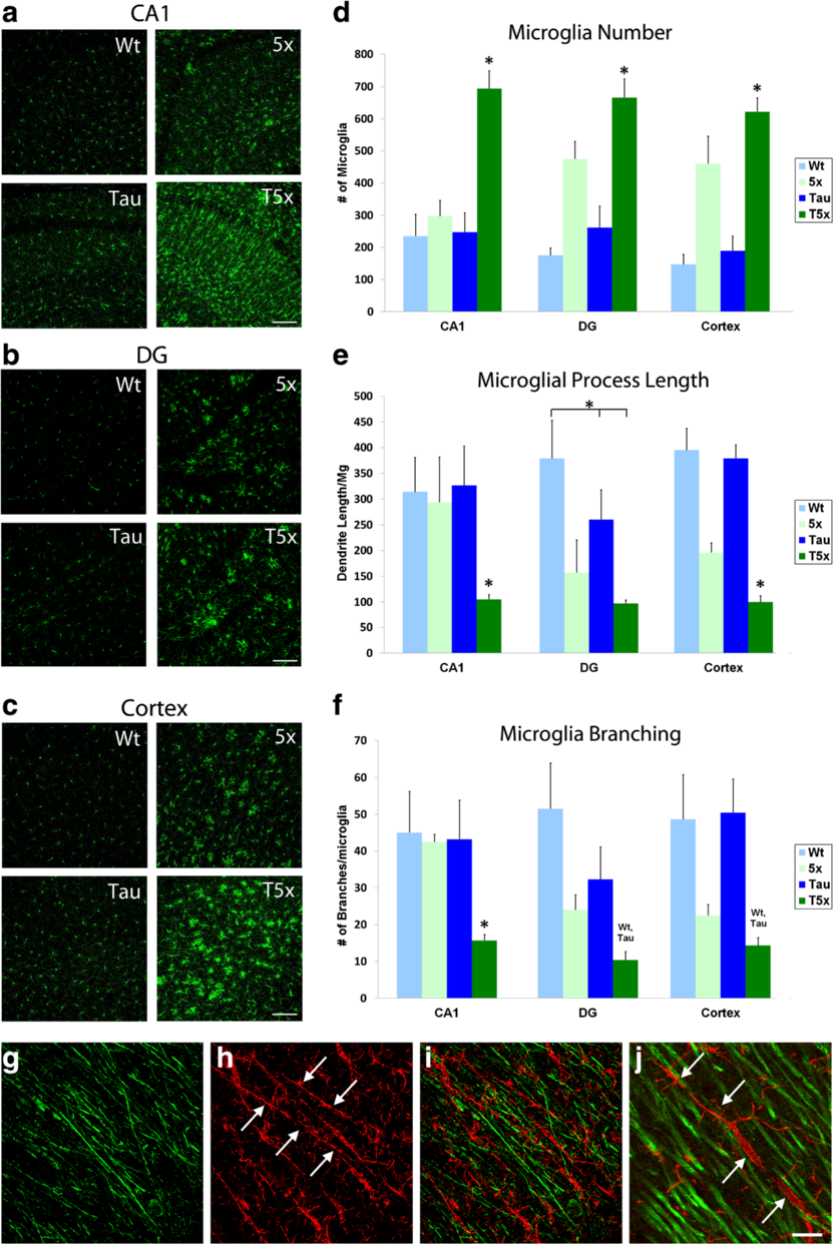
Microglial number and morphology is dramatically altered in T5x mice. Microglia were immunohistochemically labeled with IBA1 and then quantified by IMARIS bitplane analysis of confocal Z-stacks to determine the effects of Aβ, and tau pathology on microglial number and morphology. Analysis was performed within the CA1 (**a**) and dentate gyrus (**b**) of the hippocampus and the parietal association cortex (**c**), revealing a significant 2–3 fold increase in microglial number in T5x versus WT mice in each of these regions (*p* < 0.0001). T5x mice exhibited a similar 2-fold increase in microglial number relative to Thy-Tau22 mice in all 3 regions (**d**). Within CA1, T5x microglial number was also substantial higher than 5xfAD littermates (*p* < 0.0001). However, T5x mice exhibited a more subtle increase in comparison to 5xfAD mice within the dentate gyrus (*p* = 0.02) and cortex (*p* = 0.07). **e** Automated quantification of microglial process length (**e**) and branching (**f**) also revealed several significant differences withT5x microglia exhibit significantly shorter process length and decreased microglial branching compared to all other genotypes in CA1 (*p* < 0.01) and to Thy-Tau22 and WT groups within the dentate gyrus and cortex, (*p* < 0.05). **h**-**k** Examination of microglia in hippocampus CA1 revealed a unique population of rod-like microglia in T5x mice. β3-tubulin immunoreactivity (green, **g**) were associated with a microglial response, IBA-1 labeled microglia (red, **h**) were imaged within the stratum radiatum. Interestingly, this examination revealed a very specific pattern of microglial morphology within T5x mice that was not present in any of the other three genotypes. The appearance of elongated, linearly organized microglia is reminiscent of highly activated ‘rod-like’ microglia that are found in association with neurodegenerative changes [11, 87]. Thus, the microglia appear to mount a very specific response to hippocampal dendritic degeneration within T5x mice. Data are represented as mean ± SEM, *n* ≥ 8 mice/group. * Indicates *p* < 0.05 for both ANOVA and Fisher’s protected least-significant difference (PLSD) post hoc tests with significance versus all other groups, whereas *over a bar indicates significance between 2 or 3 particular groups. Scale Bar = 100 μm in (**a**-**c)**, 30 μm in (**g**-**i)**, and 10 μm in (**j**)

Next, we sought to determine the effect of amyloid and tau on microgliosis as microglia are considered the predominant immune cells of the brain, are tasked with the bulk phagocytosis of CNS material including beta-amyloid, and are known to play a prominent role in AD pathogenesis [1, 48, 50, 64]. To investigate how microglia are affected by AD pathology in T5x mice, we quantified microglial number and characterized their morphology within the CA1, the dentate gyrus (DG), and the parietal association cortex (PAC) from all four genotypes (Fig. 4a, b, c, respectively). Our analysis revealed a more than two-fold increase in the number of microglia (IBA1^+^) within T5x mice compared to the other genotypes in CA1 (*p* < 0.0001) and this increase was similarly observed within the DG and PAC (Fig. 4a, d). Activated pro-inflammatory microglia are typified by short processes and decreased branching [60]. In T5x mice, microglia process length per microglia was significantly decreased compared to all other genotypes, in all three analyzed regions: CA1, DG, and PAC (Fig. 4e, ANOVA and PLSD *p* < 0.05). T5x mice also displayed significant decreases in microglial branching per microglia compared to all genotypes, in each of the same three brain regions (Fig. 4 f, ANOVA and PLSD, *p* < 0.05). 5xfAD microglia also exhibited significantly shorter processes and decreased branching compared to WT and Thy-Tau22 in PAC and DG, supporting previous studies demonstrating that amyloid accumulation can drive microglia activation [12, 55]. However, T5x microglia exhibited shorter processes and less process branching in CA1 relative to their age-matched 5xfAD counterparts-suggesting that tau plays a synergistic role with amyloid in the modulation of microglia activation state and that the dramatic increase in tau pathology in T5x mice further exacerbates microgliosis.

Additional evidence of tau-mediated microglial activation was observed by examination of the morphology of microglial populations within CA1 of the hippocampus-an area that exhibits abundant tauopathy [16, 72]. T5x brain sections labeled for Beta-III tubulin (B3T) and IBA1 revealed a distinct microglia population uniquely surrounding the dendrites of pyramidal neurons (Fig. 4h-k). These microglia are characterized by long rod-like morphology with shortened processes and were observed in T5x mice but not 5xfAD, Thy-Tau22, or WT littermates (Fig. 4a). Rod-shaped microglia of similar morphology have recently been reported to accumulate in areas of acute neuronal injury and axonal degeneration, further supporting the notion that accumulating tau pathology further alters microglial activation in T5x mice [82, 87].

### Amyloid plaque burden is significantly reduced in T5x mice versus 5xfAD littermates

**Fig. 5:**
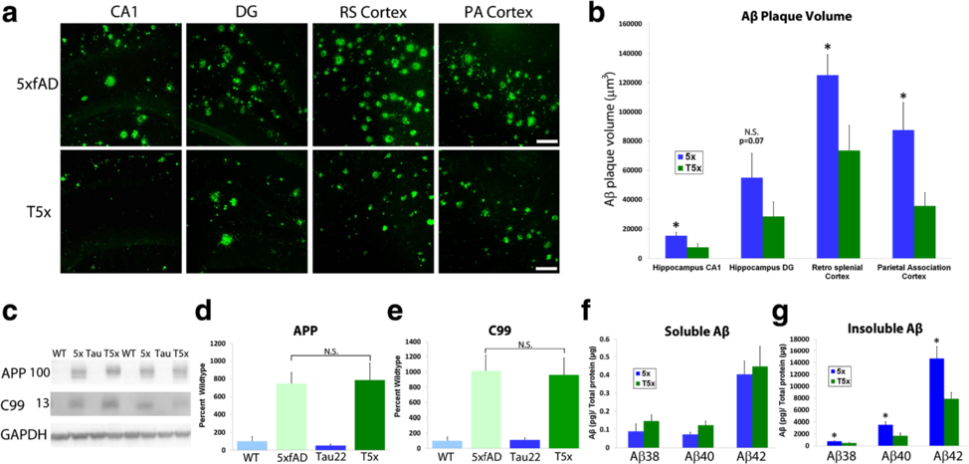
T5x mice exhibit decreased amyloid burden compared to 5xfAD littermates. **a** Amyloid deposition was quantified using immunofluorescent labeling of Aβ (82E1) followed by confocal Z-stack imaging and IMARIS bitplane software analysis within CA1 of the hippocampus (CA1) the dentate gyrus (DG), retrosplenial cortex (RSC), and parietal association cortex (PAC). **b** Amyloid plaque volume was significantly decreased in T5x mice relative to 5xfAD littermates in CA1 (*p* = 0.03), RSC (*p* = 0.04), and the PAC (0.03), whereas a nonsignificant reduction was observed in the dentate gyrus (*p* = 0.07). **c** To determine whether the observed reduction in Aβ pathology resulted from a change in APP transgene expression, human full-length APP (6E10; ~100 kDA) and C99 (6E10; ~13 kDA) was examined and quantified by western blot, revealing no differences between T5x and 5xfAD groups (**d**, **e**). Next, levels of soluble and insoluble Aβ38, 40 and 42 were examined using a Mesoscale Devices (MSD) multiplex ELISA. Interestingly, while soluble levels of Aβ where unchanged between T5x and 5xfAD groups (**f**), levels of insoluble Aβ38, 40 and 42 were significantly decreased in T5x mice relative to 5xfAD littermates (**g**). Data are represented as mean ± SEM, *n* ≥ 9 mice/group. * indicates *p* < 0.05 for both ANOVA and Fisher’s protected least-significant difference (PLSD) post hoc tests between T5x and 5x groups for that brain region or Aβ species. Scale Bar = 50 μm

Although the effects of amyloid on tau hyperphosphorylation have been extensively studied, far fewer reports have examined whether tau pathology could potentially influence amyloid burden. While we hypothesized that no differences in Aβ plaque pathology would be detected between T5x and 5xfAD littermates, changes in microgliosis suggested that microglial interactions with Aβ could potentially be altered. We therefore examined Aβ plaque load using IMARIS 3-D volumetric quantification. Surprisingly, this analysis revealed a ~50 % reduction in Aβ plaque volume within multiple brain regions of T5x versus 5xfAD mice (Fig. 5a-b, *p* < 0.05). Amyloid plaque deposition was significantly lower in the CA1 of the hippocampus, the retrosplenial cortex (RC), and parietal association cortex (PAC) of T5x versus 5xfAD littermates (ANOVA and PSLD *p* < 0.05). Within the dentate gyrus of the hippocampus, T5x mice exhibited a non-significant trend towards decreased plaque volume (*p* = 0.07, Fig. 5b).

To examine whether the decrease in amyloid burden resulted from a down-regulation of the APP transgene, APP soluble protein levels were quantified by Western blot analysis in both the cortex (Fig. 5c-e) and hippocampus (data not shown). Our results revealed no changes in APP holoprotein (*p* = 0.81) and also no change in C99, the C-terminal fragment produced by beta-secretase cleavage of APP (*p* = 0.86) between T5x and 5xfAD littermates. To determine whether alterations in Aβ might be influenced by changes in endogenous amyloidogenic enzymes we also examined the mRNA expression of BACE1, BACE2, ADAM10, PSEN1, and PSEN2 and found no significant differences across genotypes (Additional file 5: Figure S5). These data therefore support the notion that changes in Aβ load may be mediated by alterations in Aβ clearance mechanisms, rather than altered Aβ production.

**Fig. 6:**
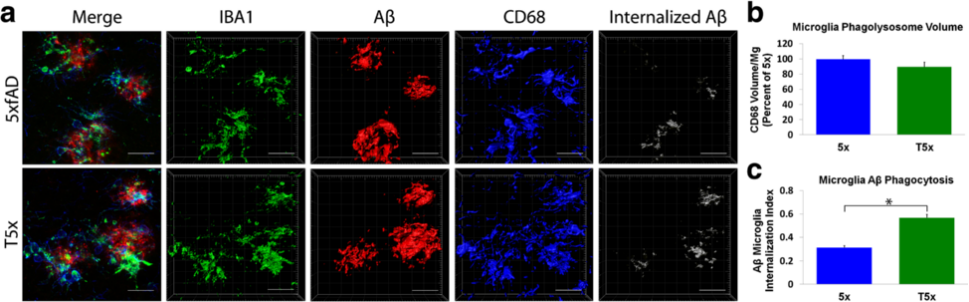
T5x microglia phagocytose more Aβ than their 5xfAD counterparts. (**a**) Equivalent 7 month old T5x and 5xfAD sections were labeled for microglia (IBA1, green), Aβ (82E1, red), and microglial phagolysosomes (CD68, blue). IMARIS bitplane software rendering of confocal Z-stacks was then used to calculate the volume of phagolysosomes within microglia and then the volume of internalized Aβ within those CD68^+^ microglial phagolysosomses, depicted in greyscale in A. Together, this analysis demonstrate that although T5x and 5xfAD microglia exhibit no differences in phagolysosome volume per microglia (**b**), the amount of Aβ within the phagolysosomes of T5x microglia was significantly increased (**c**) versus 5xfAD microglia. Data are represented as mean ± SEM, normalized to % of 5xfAD group, *n* ≥ 9 mice/group. * Indicates *p* < 0.05 for both ANOVA and Fisher’s protected least-significant difference (PLSD) post hoc tests. Scale Bar = 20 μm

To further validate the observed effects on Aβ plaque load we used a Mesoscale Discovery (MSD) V-Plex Aβ Peptide ELISA to quantify both soluble and insoluble levels of Aβ38, Aβ40, and Aβ42. MSD analysis of soluble cortical fractions revealed no significant differences between T5x and 5xfAD littermates for Aβ38 Aβ40, or Aβ42, consistent with the notion that production of soluble Aβ is unchanged (Fig. 5 f). In contrast, levels of insoluble Aβ that contribute to fibrillar Aβ plaques exhibited a significant ~2-fold decrease in T5x Aβ 38 (*p* = 0.03), Aβ40 (*p* = 0.01) and Aβ42 (*p* = 0.02) versus 5xfAD mice (Fig. 6g). Thus, in T5x mice, there is a surprising two-fold decrease in insoluble Aβ species and total plaque volume when compared to 5xfAD littermates.

### T5x microglia exhibit increased Aβ phagocytosis

While greater microglial numbers alone could potentially promote Aβ clearance, the observed changes in T5x microglial morphology and decreased brain IL-10 levels might also indicate increased microglial phagocytic capacity that could further contribute to Aβ reduction. Altered brain cytokine profiles indicate changes in microglia activation state, which can influence amyloid phagocytosis [23, 68]. For example, decreased IL-10 levels is associated with increased microglial Aβ phagocytosis [40, 54]. To test the hypothesis that reduced Aβ in T5x mice is the result of increased phagocytic capacity of T5x microglia, microglial Aβ internalization was assessed using volumetric IMARIS analysis in matching brain sections of T5x and 5xfAD mice. Microglia were identified by IBA1 immunoreactivity and Aβ phagocytosis was determined by co-localization of Aβ (82E1) and CD68 which is only present in microglial phagolysosomes (Fig. 6a). While the total volume of phagolysosome labeling per microglia was not significantly different between 5xfAD and T5x littermates (Fig. 6b, *p* = 0.18), quantification of the proportion of Aβ internalized within CD68^+^ phagolysosomes was significantly different between T5x and 5xfAD groups. In fact, this analysis revealed a nearly 30 % increase in the ability of T5x microglia to internalize Aβ versus 5xfAD littermates (Fig. 6c, *p* = 0.01). Taken together, these data sug\gest that increases in tau pathology and neuroinflammation can affect microglial Aβ phagocytosis, leading to a profound increase in amyloid clearance and reduction of plaque load in T5x mice.

## Discussion

Although the notion that amyloid increases tau pathology is well established, very few studies have in contrast examined whether a reciprocal relationship between tau and the development of amyloid pathology might exist. Beginning in 2001, two seminal studies demonstrated that Aβ could exacerbate the development of tau pathology in transgenic AD tau models [24, 47]. Subsequently, a similar causal relationship between Aβ and tau pathology was established in several other AD models [13, 17, 39, 62]. Even in the absence of the expression of a human tau transgene, many AD transgenic mice exhibit some degree of Aβ-induced tau hyperphosphorylation, a prerequisite for the development of neurofibrillary tangles. Likewise, in humans, triplication of APP, either in some family pedigrees or in trisomy 21, leads to elevated levels of Aβ and the development of tau pathology [53, 79]. Independent of Aβ, alterations in Presenilin-1 have also been shown to increase tau hyperphosphorylation in transgenic mice expressing mutant tau [14, 78]. Thus, it is quite likely that the presence of a mutant PS1 transgene in 5xfAD influences tau phosphorylation both directly and indirectly by enhancing Aβ42 generation.

As expected, the accumulation of Aβ in T5x mice leads to a dramatic increase in tau hyperphosphorylation and increased accumulation of neurofibrillary tangle pathology within the hippocampus and neocortex. Yet surprisingly, T5x mice also exhibited a 50 % reduction in amyloid plaque burden and insoluble Aβ species versus 5xfAD littermates. Although the expression of both the APP and tau transgenes are driven by the Thy1 promoter, the expression of APP in T5x mice was unchanged and therefore, decreased Aβ levels were not the result of altered transgene expression. Furthermore, levels of soluble Aβ and APP processing enzymes were unaffected; suggesting that the observed decreases in plaques and insoluble Aβ was not due to effects on APP processing.

However, the combination of amyloid and tau pathology did produce a dramatic effect on neuroinflammatory processes, included increased microgliosis and astrogliosis in T5x versus 5xfAD or Thy-Tau22 littermates. Therefore, we examined whether changes in specific cytokines could explain the increase in neuroinflammation. Our data show that the combined presence of amyloid and tau pathology exacerbates the levels of both TNF-α and IL-2 (Fig. 4). In contrast, the observed decreases in IL-1β, IL-5, and IL-6 in T5x mice versus 5xfAD littermates may reflect the predominant influence of amyloid on the release and maturation of certain pro-inflammatory cytokines. Although IL-1β has been shown to increase tau phosphorylation, amyloid has also been reported to promote astrocytic and microglial release of IL-1β [20, 36, 40, 74]. Thus, while one may expect elevated IL-1β in T5x mice in association with increased tau hyperphosphorylation [49], the influence of reduced amyloid burden on IL1β induction may take precedence over effects caused by changes in tau. Therefore, it may be the case that IL-1β, IL-5 and IL-6 are less elevated in T5x than 5xfAD mice as a consequence, rather than a cause of reduced amyloid burden. However, future studies that examine the progression of pathology and inflammation in T5x across multiple ages will likely help to resolve these questions.

TNF-α, a pro-inflammatory cytokine that is released by activated microglia was one of the most upregulated cytokines observed in T5x mice [41, 55]. Although TNF-α does not directly increase microglial proliferation, TNF-α promotes astrocyte proliferation and GM-CSF release, which in turn, can stimulate microglial proliferation [6, 45, 73]. Taken together, elevated astrocyte numbers and a ~5-fold increase in TNF-α in T5x mice relative to 5xfAD and Thy-Tau22 littermates suggests that the combined stress of amyloid and tau pathology work synergistically to promote both astrocytic and microglial proliferation. Furthermore, the significant decrease in IL-10 expression in T5x mice suggested that microglial phagocytosis of Aβ may also be altered as recent studies have shown that deletion of IL-10 can enhance microglial Aβ phagocytosis [29]. Our finding that T5x mice exhibit a similar decrease in IL-10 relative to Thy-Tau22 and 5xFAD littermates suggest that decreased IL-10 is a result of the combined stress of tau and amyloid pathology. Subsequent analysis confirmed that T5x microglia indeed have increased Aβ phagocytosis, providing further support that tau pathology contributes to microglial regulation of amyloid. It should be noted that while our results support strong interactions between tau pathology and microglial activation, the exact temporal relationship between the two is still to be determined. Additional longitudinal studies will be needed to determine whether accumulation of tau pathology first promotes microglial activation or whether changes in microglia precede and drive increases in tau hyperphosphorylation.

While cytokines are often determined by the extent of AD pathology, they can also control the progression of the disease through the modulation of microglial response. The influence of microglia in AD has received growing attention with the recent discovery of genetic risk polymorphisms in several microglial-enriched genes [15]. Some of these risk factor genes such as CD33 have themselves been implicated in microglial Aβ phagocytosis [25, 81]. Thus, the role of microglia in limiting Aβ pathology continues to garner new attention. Microglia have long been implicated in the modulation of tau hyperphosphorylation and misfolding through cytokine-mediated neuroinflammatory activation of tau kinases [49, 84]. More recently, studies have shown that the misfolding of tau can promote microglial activation [88], and tau oligomers and fibrils have been found to interact directly with microglia [56]. In addition, microglia have also recently been implicated in the propagation of tau within the brain [4]. The fact that microglia exhibit the capacity to directly phagocytose the same tau oligomers that can promote their activation, highlights the possibility that tau pathology is not only exacerbated by microglial activation, but likely in turn modulates microglial responses within the brain [52]. We reasoned that changes in cytokine levels observed in T5x mice would affect microgliosis and our current findings suggest that tau and amyloid pathology in T5x mice synergistically recruit a robust microglial response. Significantly increased activated amoeboid microglia in T5x mice versus Thy-Tau22 littermates suggests that amyloid pathology is the strongest determinant of microglial activation. Amyloid holds an advantage over predominately intracellular tau aggregates in its potential influence over microglial activation as evident of amyloid soluble oligomers released from abundant extracellular plaques [55, 70, 77]. However, our findings of increased microglial activation, phagocytosis, and numbers in T5x mice, suggest that tau pathology can also contribute to or further modulate microglia activation state.

The remaining question is exactly how development of tau pathology influences the neuroinflammatory response. One possibility is that amyloid driven tau hyperphosphorylation and subsequent release of intracellular factors and tau oligomers from degenerating neurons leads to altered microglial activation states, potentially increasing Aβ phagocytosis and thereby providing a somewhat beneficial response. Previous studies have reported the activation of microglia by the release of apoptotic factors and intracellular molecules such as ADP or ATP [21, 37, 58]. When we examined T5x mice for dendritic degeneration, we observed a reduction in hippocampal Beta-III tubulin staining in T5x mice compared to transgenic and WT littermates (Additional file 6: Figure S6). Notably, hippocampal regions displaying the significant reduction in Beta-III tubulin were accompanied by the previously described rod microglia (Fig. 4 g-j) [80]. Although not extensively characterized, rod microglia have been found in human AD cases and exhibit abnormally frequent interactions with synaptic clefts [46, 59, 82]. T5x rod-like microglia are similar in morphology to recently described rod-microglia which appear highly activated and respond to diffuse neuronal injury [87]. The association between reduced β3-Tubullin density and rod microglia presence therefore suggests that degenerating dendrites and subsequent release of intracellular factors such as ADP may contribute to the altered microglia activation state in T5x mice. However, further studies are clearly needed to elucidate the exact mechanisms by which tau synergizes with Aβ to modulate microglial activation.

It is important to acknowledge that our findings both corroborate and differ from other recently examined amyloid-tau crosses. We report a significant reduction in amyloid plaque burden and insoluble Aβ in T5x mice relative to 5xfAD littermates that appears to be mediated via increased microglial-mediated Aβ clearance. Using a similar model, one group has shown that 5xfAD crossed with Tg30 tau mice likewise exhibit decreased Aβ pathology, although the potential role of microglia in this finding was not examined [35]. In contrast, two other studies crossed 5xfAD mice with other tau models and reported no change in Aβ plaques by optical densitometry, although only 3 or 4 mice per group were compared versus group sizes of 9–10 mice for the current Aβ ELISA analysis [75]. Yet, one of these studies nevertheless showed a non-significant 45 % reduction in Aβ plaque load at 3 months of age. It is therefore quite possible that the use of 3D plaque volume quantification, ELISA, or a larger sample size would have revealed a similar significant reduction in Aβ to that observed in the current study. In another study, Tg2576 APP mice were crossed with mutant tau VLW mice. Surprisingly, this study reported a significant increase in amyloid deposition in bigenic mice [67]. Likewise, a recent study that examined a cross between rTgTauEC and APP/PS1 mice also reported increased amyloid burden [66].

These varying reports suggest that perhaps the magnitude of tau pathology and/or microgliosis can influence the effects on Aβ pathology. In support of this notion, we find that T5x mice exhibit a dramatic increase in microglial number, shift in morphology, and change in cytokines that is not apparent in Thy-Tau22 littermates with lower levels of tau pathology (Figs. 4 and 5). Interestingly, in other studies that crossed different tau models with 5xfAD mice [35, 75], there were either no significant changes or a similar decrease in amyloid pathology. In contrast, one study reported an increase in amyloid burden when Tg2576 mice were crossed with VLW tau mice [67]. 5xfAD mice, which carry three APP and two PS1 mutations, generate considerably more amyloid pathology than Tg2576 mice and the presence of a PS1 transgene may further promote tau hyperphosphorylation both directly [14, 78] and indirectly by enhancing Aβ42 generation. Thus, differences in levels of tau, the type and magnitude of amyloid pathology, and their corresponding effects on microglial activation state might explain these discrepancies. For example, it is quite possible that the more aggregation prone Aβ42 leads to a greater increase in tau pathology and a differential activation of microglia that combine to promote microglial phagocytosis. Another possible explanation for these contrasting findings is the potential impact of background strain on pathology. In the current study we crossed congenic C57/Bl6 5xfAD mice with congenic C57/Bl6 Thy-Tau22 mice, thus background strain was identical between all four genotypes examined. In some of the contrasting studies, the cross-bred mice exhibited differential mixed background strains that include Bl6, CH3, and FVB [66] and thus variability in background genetics even between littermates could influence the development of pathology. Although our current data clearly suggests that tau pathology can reduce the accumulation of Aβ, these prior reports that in many cases corroborate our findings but in others contrast our findings clearly suggest that this remains a highly complex question that will need additional examination. For example, it is quite possible that changes in APP transport or alterations in autophagy also play a role in this process and thus studies that further examine these additional potential mechanisms are also needed.

## Conclusion

In conclusion, we have established an AD model that provides further insight into the potential reciprocal relationships between amyloid, tau and neuroinflammation. The combination of these pathologies significantly increases tau hyperphosphorylation and microgliosis, yet decreases amyloid burden. Our data also suggest that tau-induced increases in microglial number, and phagocytic activity may explain the reductions in amyloid burden. The changes observed in amyloid accumulation and microgliosis in the presence of tau pathology suggests that therapies targeting tauopathy could have increased benefit towards treating additional underlying proponents of AD. Therefore, our study adds to the growing understanding of the role of microglia in AD pathogenesis and suggests that differential activation of these cells in response to Aβ and tau pathology can have both detrimental as well as beneficial effects on disease progression.

## Additional files

Additional file 1: Figure S1. Tau hyperphoshorylation within the cortex is also increased by Aβ accumulation. (**A**) Although levels of soluble unphosphorylated human tau (HT7 60 kDA, *p* = 0.19), p-S199, and p-S201 are not significantly different between T5x and Thy-Tau22 littermates, phosphorylation of tau at the 65 kDA HT7 band and the combined phosphorylation of both 60 and 65 kDa bands as detected with AT100, AT270, and PHF1 pathological epitope antibodies is greatly increased (*p* < 0.01). (**B**) Levels of insoluble human tau (HT7), p-S199, and p-S201 within the cortex are also unchanged, but again insoluble accumulation of AT100, AT270, and PHF1 phosphorylated tau is dramatically elevated in T5x mice (*p* < 0.0001). Immunohistochemical labeling of AT8 (**C**) and PHF1 (**D**) in the cortex further demonstrate the considerable increase in tau accumulation that occurs in T5x mice. Likewise cortical labeling of MC1 at low (E) and high (F) magnification (quantified in Additional file 2: Figure S2) again reveal a significant increase in this pathological conformational tau epitope. Data are represented as mean ± SEM, normalized to % of WT group, *n* ≥ 8 mice/group. * Indicates *p* < 0.05 for both ANOVA and Fisher’s protected least-significant difference (PLSD) post hoc tests with significance versus all other groups, whereas *over a bar indicates significance between 2 or 3 particular groups. Scale Bar = 100 μm in C-E and 50 μm in F. (PDF 16547 kb) Additional files 2: Figure S2. T5x mice exhibit increased levels of Tau phosphorylation and misfolding. (**A**) The ratio of phosphorylated human tau (65 kDa) to unphosphorylated human tau (60 kDa) was quantified by western blot. Analysis revealed significantly higher 65:60 kDa HT7 ratio in T5x mice relative to Tau22 littermates, suggesting that tau hyperphosphorylation is exacerbated by the presence of Aβ in T5x mice. (**B**) Equivalent hippocampal sections were examined from 7-month old T5x and Thy-Tau22 mice and immunohistochemically labeled with the conformational-specific tau antibody; MC-1 (see Figs. 2 and 3). Quantification revealed a significant increase in the numbers of MC-1-positive neurons in hippocampus CA1 (*p* < 0.0001) and an even greater increase in MC-1-immunoreactive cells within the perirhinal/entorhinal cortex. Data are represented as mean ± SEM of optical density (O.D.), *n* ≥ 8 mice/group. * Indicates *p* < 0.05 for both ANOVA and Fisher’s protected least-significant difference (PLSD) post hoc tests. (PDF 427 kb) Additional files 3: Figure S3. Changes in tau pathology correlate closely with alterations in TNFα and IL-2. (**A**) Further demonstrating the shift in cytokines that occurs in T5x mice, IL-6 and TNFα exhibit a bimodal distribution, with high levels of TNF α but low levels of IL-6 in T5x mice (green) versus low TNFα and high IL-6 in WT (purple), Thy-Tau22 (blue), and 5xfAD (red) mice. (**B**) TNFα and IL-2 expression are very closely correlated (R^2^ = 0.925) especially in T5x mice (green), illustrating a strong concordance between these two pro-inflammatory cytokines. Both soluble (**C**) and insoluble (**D**) measures of cortical PHF-1 tau correlate well with cortical TNFα levels. Likewise, soluble (E) and insoluble (F) measures of PHF-1 tau also correlate closely with IL-2 expression. (PDF 920 kb) Additional files 4: Figure S4. T5x mice exhibit increased astrogliosis. (**A**, **B**) Immunohistochemical analysis and IMARIS quantification of GFAP-labeled astrocytes reveal elevated numbers within CA1 of the hippocampus (WT *p* = 0.13; 5x *p* = 0.03; Tau *p* = 0.04) and the parietal association cortex (WT, Tau *p* < 0.0001; 5x *p* = 0.0002), but no differences within the dentate gyrus. (**C**) Western blot analysis of GFAP in cortical fractions corroborate these findings by demonstrating a significant increase in GFAP between T5x and both WT and Thy-Tau22 groups (WT *p* = 0.006; 5x *p* = 0.10; Tau *p* = 0.002). Data are represented as mean ± SEM of optical density (O.D.), *n* ≥ 8 mice/group. * Indicates *p* < 0.05 for both ANOVA and Fisher’s protected least-significant difference (PLSD) post hoc tests with significance versus all other groups, whereas *over a bar indicates significance between 2 or 3 particular groups. Scale Bar = 100 μm. (PDF 11243 kb) Additional files 5: Figure S5. Nanostring analysis reveals no differences in mRNA expression of APP-processing enzymes. Hippocampal mRNA was isolated from each genotype and examined using a custom Nanostring panel to quantify APP-processing associated genes including murine APP, BACE1, BACE2, ADAM10, PSEN1, and PSEN2. In each case, no differences between genotype were observed. (PDF 433 kb) Additional files 6: Figure S6. The combination of Aβ and Tau pathology leads to reductions in hippocampal β3-tubulin. (**A**-**D**) To determine whether T5x mice begin to exhibit early signs of neurodegeneration, dendritic architecture was examined by β3-tubulin immunolabeling of all four genotypes. (**E**-**G**) Quantification of β3-tubulin revealed a significant reduction in T5x mice compared to WT and transgenic littermates within the pyramidal cell layer (E; *p* < 0.05), stratum radiatum (F; *p* < 0.05), and molecular layer (G; WT, Tau *p* < 0.05; 5x *p* = 0.27) of the hippocampus. Data are represented as mean ± SEM of optical density (O.D.), *n* ≥ 8 mice/group. * Indicates *p* < 0.05 for both ANOVA and Fisher’s protected least-significant difference (PLSD) post hoc tests with significance versus all other groups, whereas *over a bar indicates significance between 2 or 3 particular groups. Scale Bar = 100 μm in **A**-**D**, 30 μm in **H**-**J**, and 10 μm in **K**. (PDF 3896 kb)
