# Supplementary figures and images for: Increased tauopathy drives microglia-mediated clearance of beta-amyloid
Source: Acta Neuropathol Commun. 2016 Jun 23;4:63. doi: 10.1186/s40478-016-0336-1 (PMC4918195; doi:10.1186/s40478-016-0336-1)

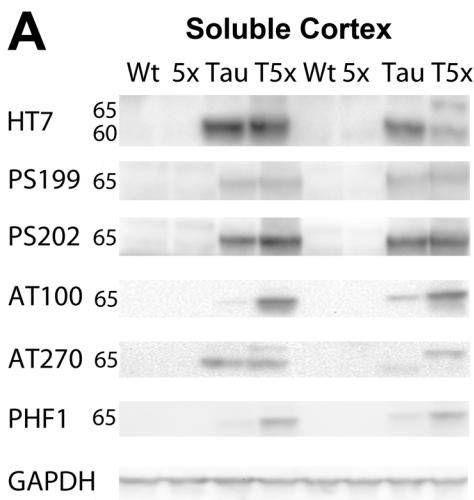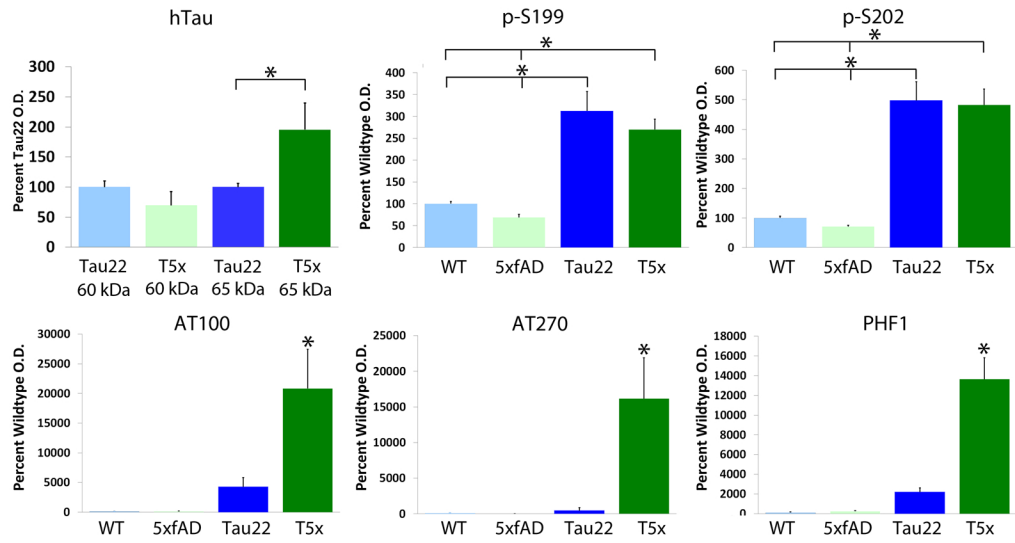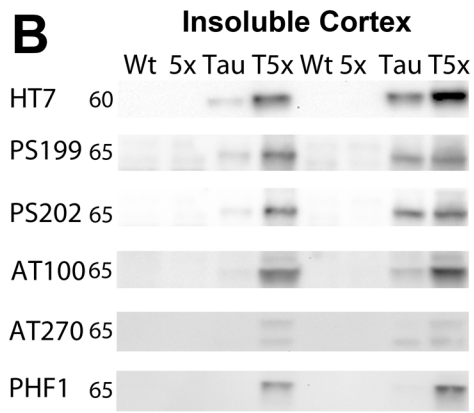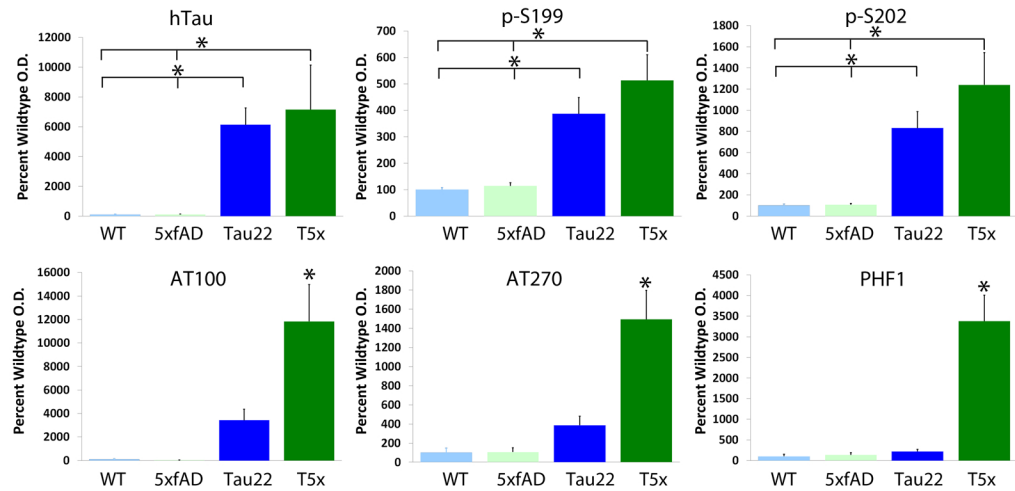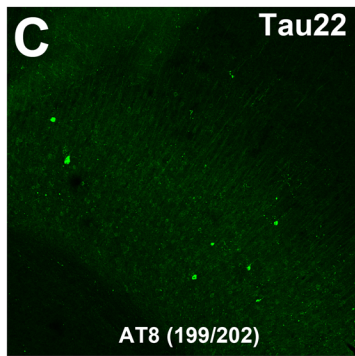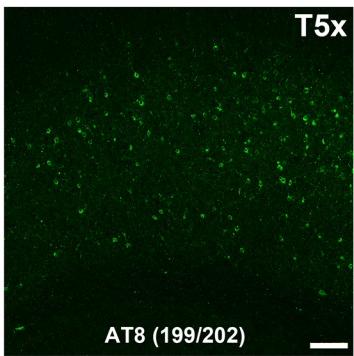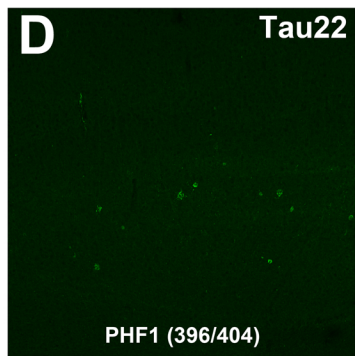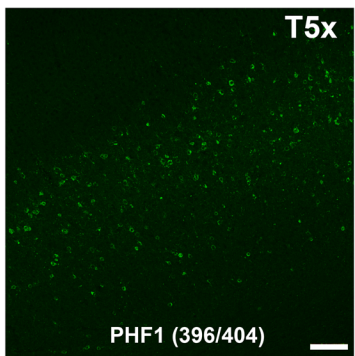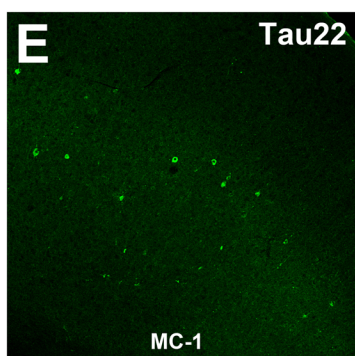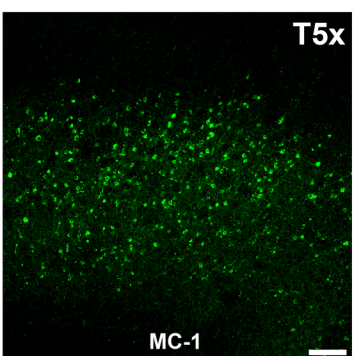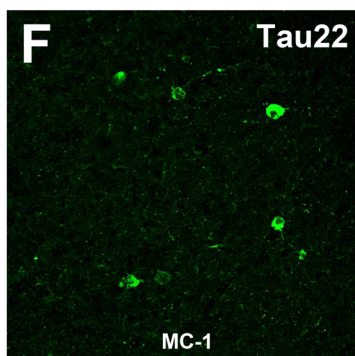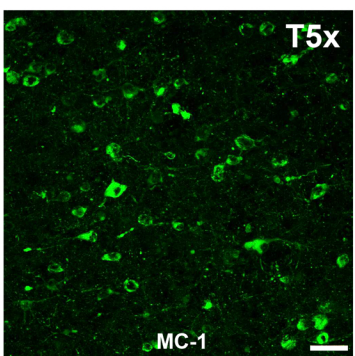

Supplement: Additional file 1: Figure S1. — Tau hyperphoshorylation within the cortex is also increased by Aβ accumulation. (A) Although levels of soluble unphosphorylated human tau (HT7 60 kDA, p = 0.19), p-S199, and p-S201 are not significantly different between T5x and Thy-Tau22 littermates, phosphorylation of tau at the 65 kDA HT7 band and the combined phosphorylation of both 60 and 65 kDa bands as detected with AT100, AT270, and PHF1 pathological epitope antibodies is greatly increased (p < 0.01). (B) Levels of insoluble human tau (HT7), p-S199, and p-S201 within the cortex are also unchanged, but again insoluble accumulation of AT100, AT270, and PHF1 phosphorylated tau is dramatically elevated in T5x mice (p < 0.0001). Immunohistochemical labeling of AT8 (C) and PHF1 (D) in the cortex further demonstrate the considerable increase in tau accumulation that occurs in T5x mice. Likewise cortical labeling of MC1 at low (E) and high (F) magnification (quantified in Additional file 2: Figure S2) again reveal a significant increase in this pathological conformational tau epitope. Data are represented as mean ± SEM, normalized to % of WT group, n ≥ 8 mice/group. * Indicates p < 0.05 for both ANOVA and Fisher’s protected least-significant difference (PLSD) post hoc tests with significance versus all other groups, whereas *over a bar indicates significance between 2 or 3 particular groups. Scale Bar = 100 μm in C-E and 50 μm in F. (PDF 16547 kb) [file 40478_2016_336_MOESM1_ESM.pdf]

**A**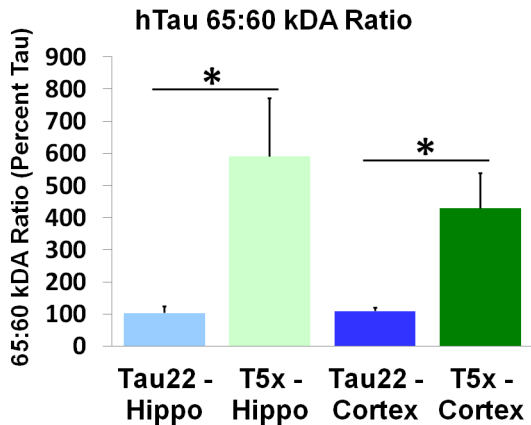**B**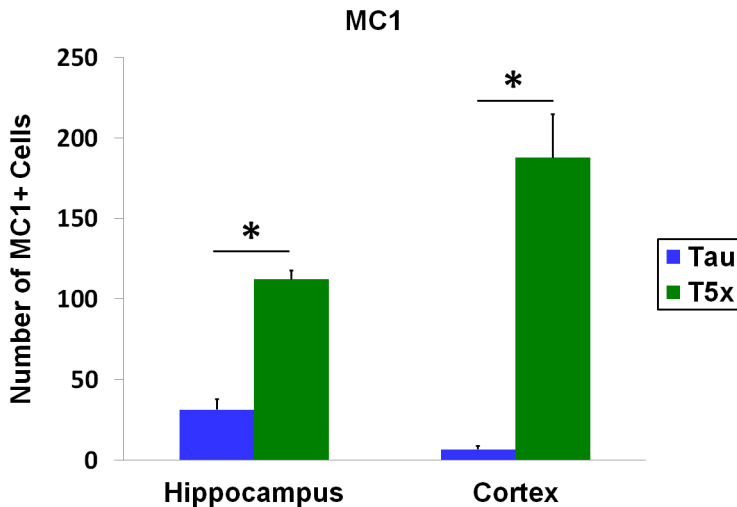

Supplement: Additional files 2: Figure S2. — T5x mice exhibit increased levels of Tau phosphorylation and misfolding. (A) The ratio of phosphorylated human tau (65 kDa) to unphosphorylated human tau (60 kDa) was quantified by western blot. Analysis revealed significantly higher 65:60 kDa HT7 ratio in T5x mice relative to Tau22 littermates, suggesting that tau hyperphosphorylation is exacerbated by the presence of Aβ in T5x mice. (B) Equivalent hippocampal sections were examined from 7-month old T5x and Thy-Tau22 mice and immunohistochemically labeled with the conformational-specific tau antibody; MC-1 (see Figs. 2 and 3). Quantification revealed a significant increase in the numbers of MC-1-positive neurons in hippocampus CA1 (p < 0.0001) and an even greater increase in MC-1-immunoreactive cells within the perirhinal/entorhinal cortex. Data are represented as mean ± SEM of optical density (O.D.), n ≥ 8 mice/group. * Indicates p < 0.05 for both ANOVA and Fisher’s protected least-significant difference (PLSD) post hoc tests. (PDF 427 kb) [file 40478_2016_336_MOESM2_ESM.pdf]

**A**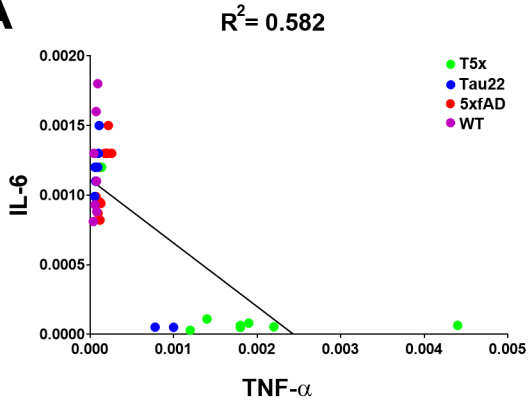**B**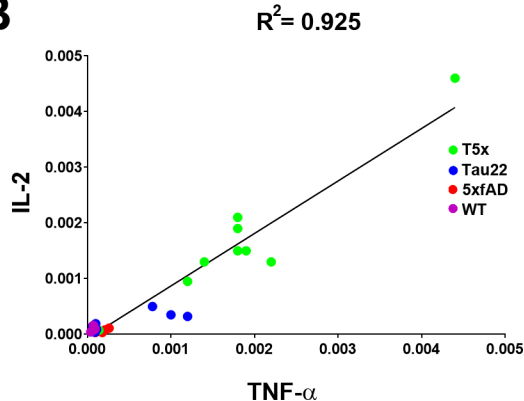**C**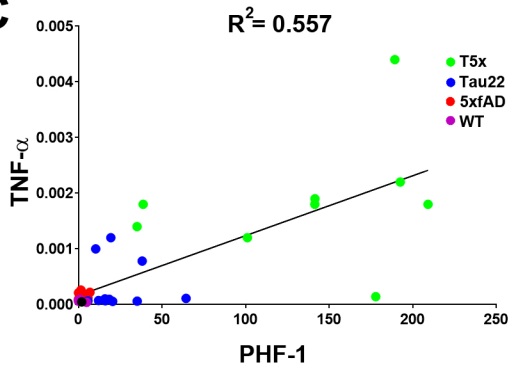**D**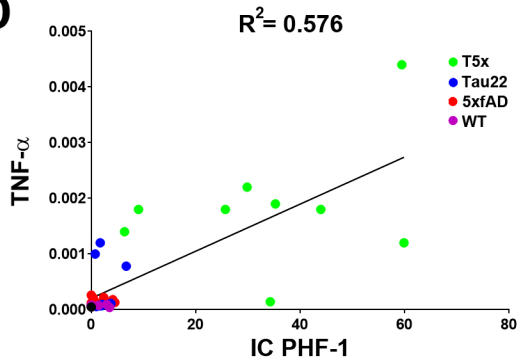**E**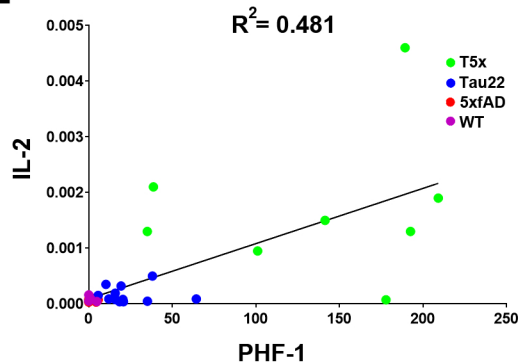**F**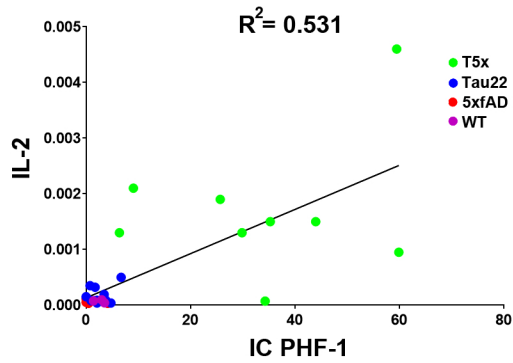

Supplement: Additional files 3: Figure S3. — Changes in tau pathology correlate closely with alterations in TNFα and IL-2. (A) Further demonstrating the shift in cytokines that occurs in T5x mice, IL-6 and TNFα exhibit a bimodal distribution, with high levels of TNF α but low levels of IL-6 in T5x mice (green) versus low TNFα and high IL-6 in WT (purple), Thy-Tau22 (blue), and 5xfAD (red) mice. (B) TNFα and IL-2 expression are very closely correlated (R2 = 0.925) especially in T5x mice (green), illustrating a strong concordance between these two pro-inflammatory cytokines. Both soluble (C) and insoluble (D) measures of cortical PHF-1 tau correlate well with cortical TNFα levels. Likewise, soluble (E) and insoluble (F) measures of PHF-1 tau also correlate closely with IL-2 expression. (PDF 920 kb) [file 40478_2016_336_MOESM3_ESM.pdf]

**A**

CA1

DG

Cortex

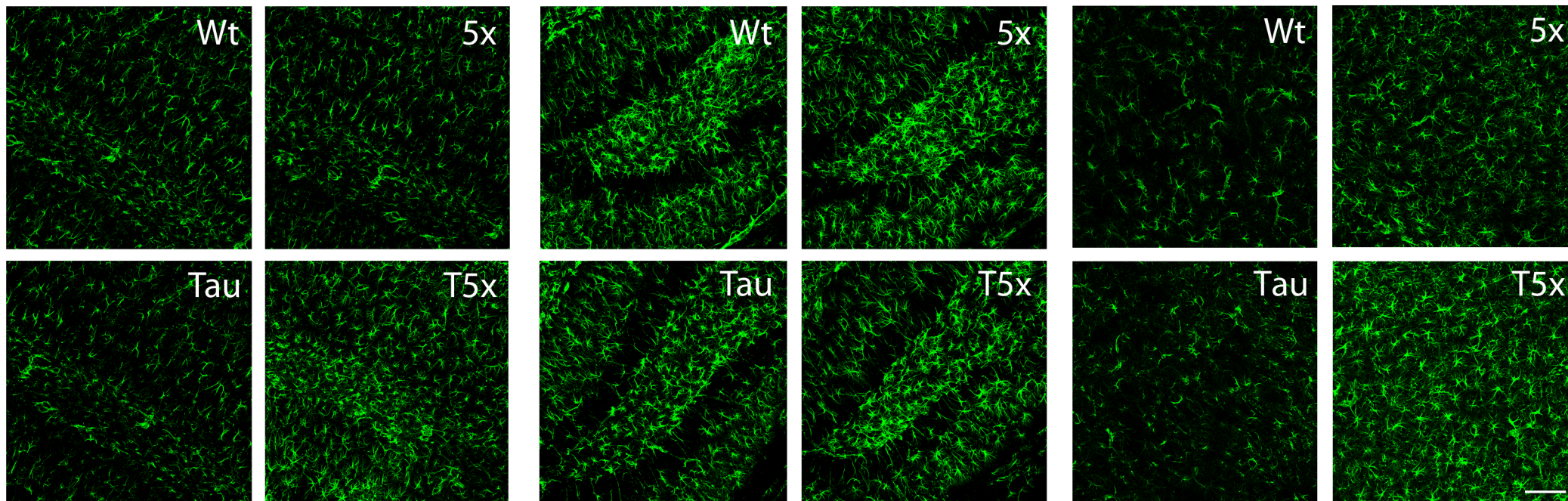**B**

Astrocyte Volume

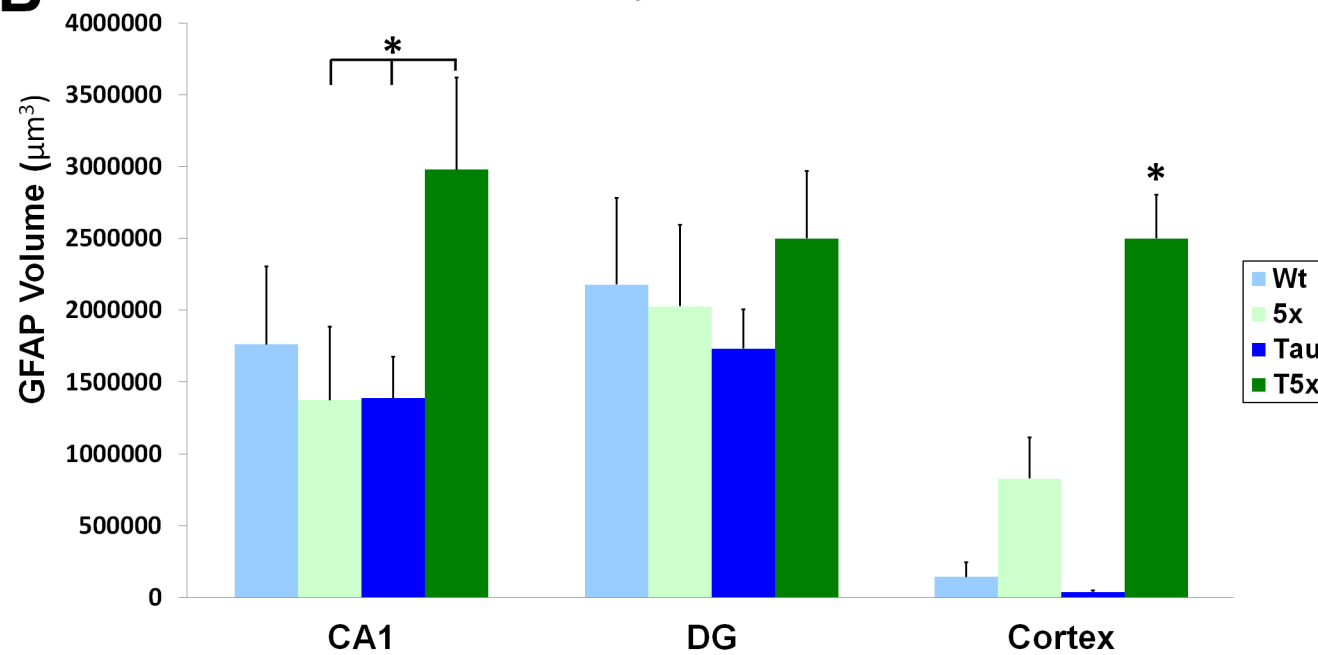**C**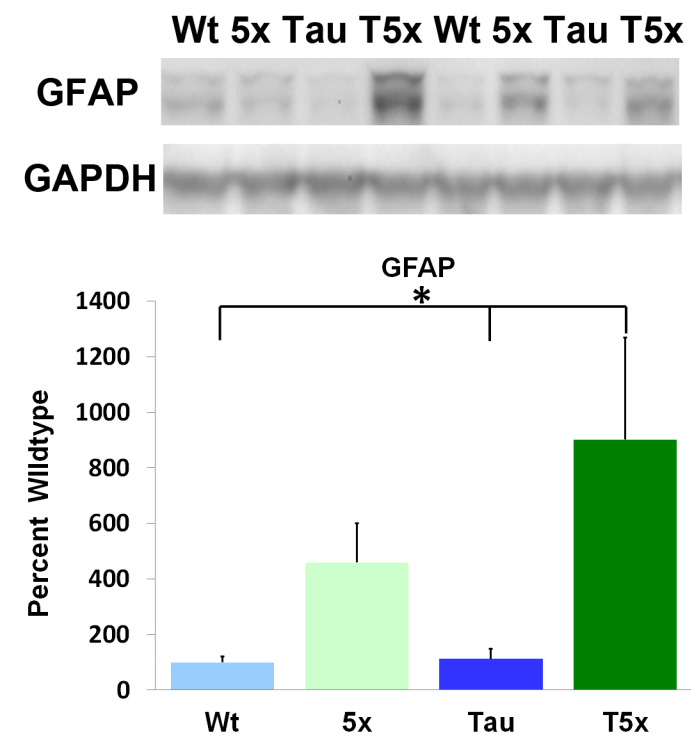

Supplement: Additional files 4: Figure S4. — T5x mice exhibit increased astrogliosis. (A, B) Immunohistochemical analysis and IMARIS quantification of GFAP-labeled astrocytes reveal elevated numbers within CA1 of the hippocampus (WT p = 0.13; 5x p = 0.03; Tau p = 0.04) and the parietal association cortex (WT, Tau p < 0.0001; 5x p = 0.0002), but no differences within the dentate gyrus. (C) Western blot analysis of GFAP in cortical fractions corroborate these findings by demonstrating a significant increase in GFAP between T5x and both WT and Thy-Tau22 groups (WT p = 0.006; 5x p = 0.10; Tau p = 0.002). Data are represented as mean ± SEM of optical density (O.D.), n ≥ 8 mice/group. * Indicates p < 0.05 for both ANOVA and Fisher’s protected least-significant difference (PLSD) post hoc tests with significance versus all other groups, whereas *over a bar indicates significance between 2 or 3 particular groups. Scale Bar = 100 μm. (PDF 11243 kb) [file 40478_2016_336_MOESM4_ESM.pdf]

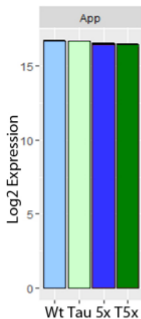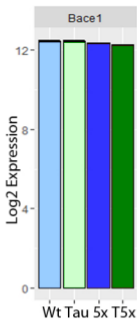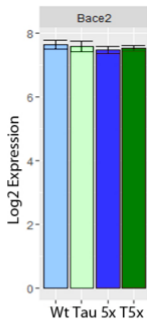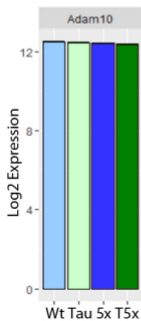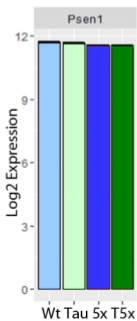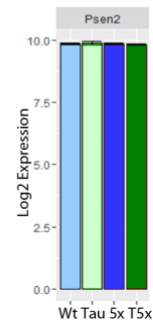

Supplement: Additional files 5: Figure S5. — Nanostring analysis reveals no differences in mRNA expression of APP-processing enzymes. Hippocampal mRNA was isolated from each genotype and examined using a custom Nanostring panel to quantify APP-processing associated genes including murine APP, BACE1, BACE2, ADAM10, PSEN1, and PSEN2. In each case, no differences between genotype were observed. (PDF 433 kb) [file 40478_2016_336_MOESM5_ESM.pdf]

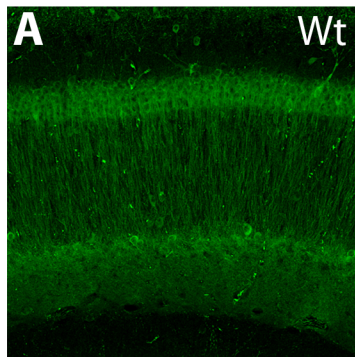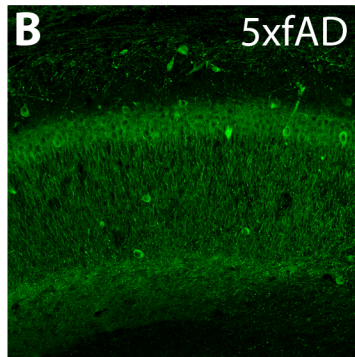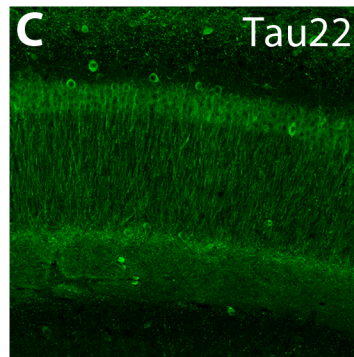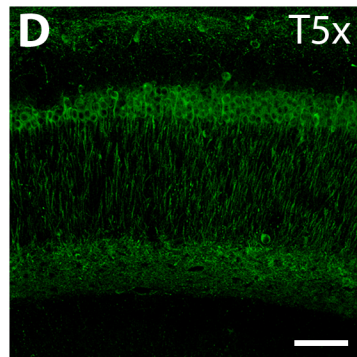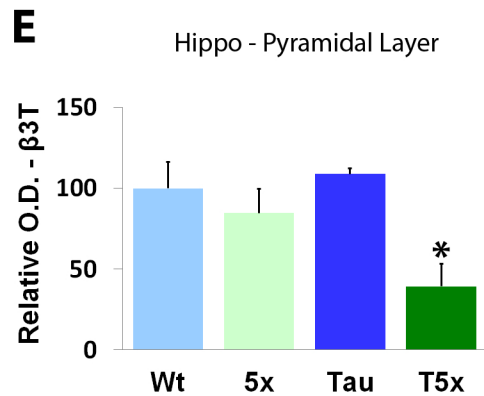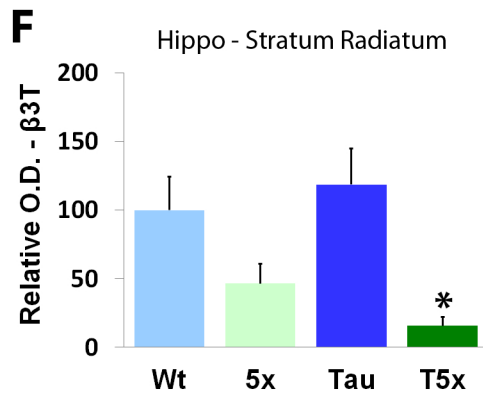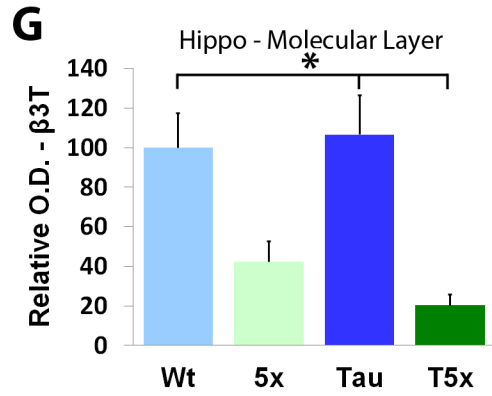

Supplement: Additional files 6: Figure S6. — The combination of Aβ and Tau pathology leads to reductions in hippocampal β3-tubulin. (A-D) To determine whether T5x mice begin to exhibit early signs of neurodegeneration, dendritic architecture was examined by β3-tubulin immunolabeling of all four genotypes. (E-G) Quantification of β3-tubulin revealed a significant reduction in T5x mice compared to WT and transgenic littermates within the pyramidal cell layer (E; p < 0.05), stratum radiatum (F; p < 0.05), and molecular layer (G; WT, Tau p < 0.05; 5x p = 0.27) of the hippocampus. Data are represented as mean ± SEM of optical density (O.D.), n ≥ 8 mice/group. * Indicates p < 0.05 for both ANOVA and Fisher’s protected least-significant difference (PLSD) post hoc tests with significance versus all other groups, whereas *over a bar indicates significance between 2 or 3 particular groups. Scale Bar = 100 μm in A-D, 30 μm in H-J, and 10 μm in K. (PDF 3896 kb) [file 40478_2016_336_MOESM6_ESM.pdf]
